# Effects of empagliflozin on left ventricular diastolic function in addition to usual care in individuals with type 2 diabetes mellitus—results from the randomized, double-blind, placebo-controlled EmDia trial

**DOI:** 10.1007/s00392-023-02164-w

**Published:** 2023-02-10

**Authors:** Jürgen H. Prochaska, Claus Jünger, Andreas Schulz, Natalie Arnold, Felix Müller, Marc William Heidorn, Rieke Baumkötter, Daniela Zahn, Thomas Koeck, Sven-Oliver Tröbs, Karl J. Lackner, Andreas Daiber, Harald Binder, Sanjiv J. Shah, Tommaso Gori, Thomas Münzel, Philipp S. Wild

**Affiliations:** 1 Preventive Cardiology and Preventive Medicine, Department of Cardiology, University Medical Center of the Johannes Gutenberg-University Mainz, Langenbeckstr. 1, 55131 Mainz, Germany; 2 Center for Thrombosis and Hemostasis (CTH), University Medical Center of the Johannes Gutenberg-University Mainz, Mainz, Germany; 3 German Center for Cardiovascular Research (DZHK), Partner Site Rhine Main, Mainz, Germany; 4 Department of Psychosomatic Medicine and Psychotherapy, University Medical Center of the Johannes Gutenberg-University Mainz, Mainz, Germany; 5 Institute of Medical Biometry and Statistics, University of Freiburg, Freiburg, Germany; 6 Department of Cardiology, University Heart and Vascular Center Hamburg, Hamburg, Germany; 7 Institute for Clinical Chemistry and Laboratory Medicine, University Medical Center of the Johannes Gutenberg-University Mainz, Mainz, Germany; 8 Department of Cardiology, University Medical Center of the Johannes Gutenberg-University Mainz, Mainz, Germany; 9 Division of Cardiology, Department of Medicine, Northwestern University Feinberg School of Medicine, Chicago, IL USA; 10 Systems Medicine, Institute for Molecular Biology (IMB), Mainz, Germany

**Keywords:** Type 2 diabetes mellitus, Heart failure, Diastolic function, Biomarkers, Clinical trial

## Abstract

**Background:**

The sodium-glucose co-transporter 2 inhibitor empagliflozin improves cardiovascular outcome in patients with type 2 diabetes mellitus (T2DM) and heart failure. Experimental studies suggest a direct cardiac effect of empagliflozin associated with an improvement in left ventricular diastolic function.

**Methods:**

In the randomized, double-blind, two-armed, placebo-controlled, parallel group trial EmDia, patients with T2DM and elevated left ventricular *E*/*E*´ ratio were enrolled and randomized 1:1 to receive empagliflozin 10 mg/day versus placebo. The primary endpoint was the change of left ventricular *E*/*E*´ ratio after 12 weeks of intervention.

**Results:**

A total of 144 patients with T2DM and an elevated left ventricular *E*/*e*´ ratio (age 68.9 ± 7.7 years; 14.1% women; *E*/*e*´ ratio 9.61[8.24/11.14], left ventricular ejection fraction 58.9% ± 5.6%). After 12 weeks of intervention, empagliflozin resulted in a significant higher decrease in the primary endpoint *E*/*e*´ ratio by − 1.18 ([95% confidence interval (CI) − 1.72/− 0.65]; *P* < 0.0001) compared with placebo. The beneficial effect of empagliflozin was consistent across all subgroups and also occurred in subjects with heart failure and preserved ejection fraction (*n* = 30). Additional effects of empagliflozin on body weight, HbA1c, uric acid, red blood cell count, hemoglobin, mean corpuscular hemoglobin, and hematocrit were detected (all *P* < 0.001). Approximately one-third of the reduction in *E*/*e*´ by empagliflozin could be explained by the variables examined.

**Conclusions:**

Empagliflozin improves diastolic function in patients with T2DM and elevated end-diastolic pressure. Since the positive effects were consistent in patients with and without heart failure with preserved ejection fraction, the data add a mechanistic insight for the beneficial cardiovascular effect of empagliflozin.

**Trial registration:**

Clinicaltrials.gov, unique identifier: NCT02932436.

**Graphical abstract:**

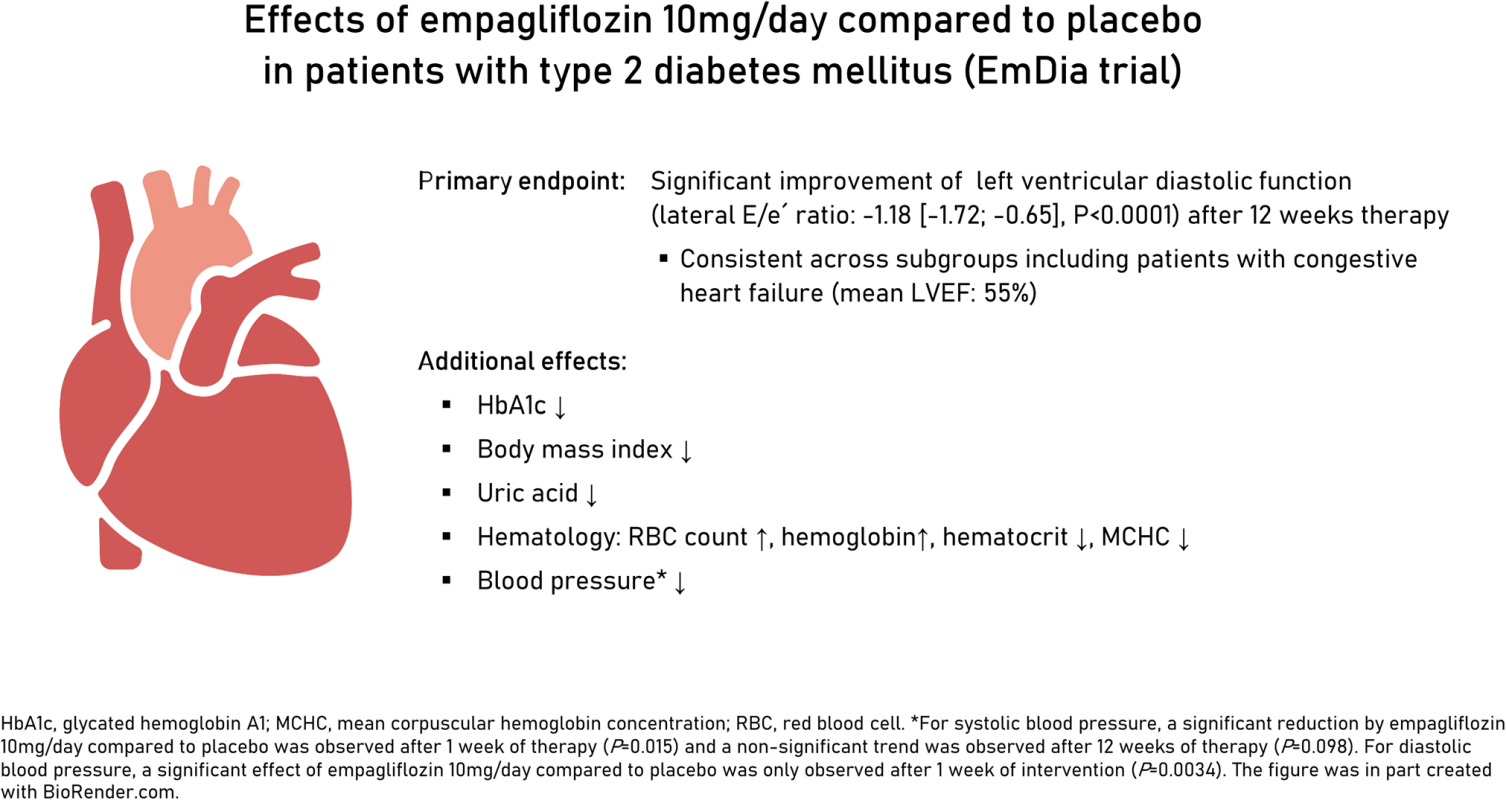

**Supplementary Information:**

The online version contains supplementary material available at 10.1007/s00392-023-02164-w.

## Introduction

Diabetes mellitus is an epidemic disease affecting more than 460 million people worldwide [1]. In recent years, inhibitors of the sodium-dependent glucose co-transporter 2 (SGLT2) have received marketing approval as antidiabetic agents following positive cardiovascular outcome trials: in the EMPA-REG Outcome trial, the SGLT2 inhibitor empagliflozin reduced the risk of cardiovascular death, non-fatal myocardial infarction or non-fatal stroke by 14% compared with placebo, mainly due to a strong reduction in cardiovascular death [2]. Analysis of the cumulative incidence demonstrated a separation between both groups that was detectable as early as two months after initiation of therapy. In a secondary analysis, empagliflozin was associated with a 35% reduction of hospitalization for heart failure, also observed immediately after treatment initiation, suggesting a very early effect on the failing heart [3]. Together with evidence from several other cardiovascular outcome trials, accumulating evidence to a paradigm shift in antidiabetic therapy. Clinical trials are have recently been completed reporting beneficial efficacy and safety of empagliflozin compared to placebo in patients with heart failure with and without diabetes mellitus [4, 5].

The specific mechanisms mediating the beneficial effects of empagliflozin on cardiovascular outcome remain controversial [6]. Previous studies have suggested that the effect on the heart may be related to an improvement in left ventricular diastolic function [7, 8]. However, comprehensive evidence from randomized studies in humans that investigate the effects of empagliflozin on cardiac function along with humoral cardiac, metabolic and hematological biomarkers is currently lacking.

The EmDia trial was designed to evaluate the effect of empagliflozin compared to placebo on left ventricular diastolic function in subjects with type 2 diabetes mellitus and elevated left ventricular end-diastolic pressure in a randomized, double-blind controlled clinical trial combined with comprehensive clinical and molecular phenotyping.

## Methods

### Trial design

The EmDia trial is a randomized, double-blind, two-armed, placebo-controlled, parallel group, investigator-initiated study of phase IV. The University Medical Center of the Johannes Gutenberg-University Mainz conducted the single-center trial as study sponsor. All study documents were approved by the local ethics committee and the data protection officer prior to study initiation. All study participants provided informed written consent, and study procedures have been performed in line with the principles outlined in the Declaration of Helsinki and the recommendations for Good Clinical Practice. The trial was registered at clinicaltrials.gov with the unique identifier: NCT02932436 (EudraCT number: 2016-001264-11). The rationale and design of the trial have been described in detail recently [9].

### Patient enrolment and randomization

The main inclusion criteria of the EmDia trial were: (i) age from 18 to 84 years, (ii) diagnosis of type 2 diabetes mellitus with stable glucose-lowering background therapy and/or dietary treatment for at least 12 weeks, (iii) HbA1c ≥ 6.5% and ≤ 10.0% in subjects on antidiabetic background therapy or HbA1c ≥ 6.5% and ≤ 9.0% for drug-naïve subjects with dietary treatment, and (iv) prevalent left ventricular diastolic dysfunction defined as left ventricular lateral *E*/*E*´ ratio ≥ 8 in transthoracic echocardiography. Main exclusion criteria were impaired renal function, defined as estimated glomerular filtration rate (eGFR [10]) < 45 ml/min/1.73 m^2^ of body-surface-area or end-stage renal failure or dialysis or uncontrolled hyperglycemia with a glucose level > 240 mg/dl (> 13.3 mmol/L) after overnight fast.

Patients who met all inclusion criteria and none of the exclusion criteria were randomized 1:1 to the intervention or control group at the baseline visit. Block-randomization including sex-stratification was performed by an independent institution (Interdisciplinary Center for Clinical Trials (IZKS), Mainz, Germany). During the 12-week trial period after randomization, patients received empagliflozin at a dose of 10 mg per day or an identical placebo in addition to the concomitant medication.

### Trial procedures

At the dedicated study center, patients received a highly standardized 5-h clinical and medical technical examination from October 2016 to June 2020. Trained and certified medical assistants performed all procedures according to standard operating procedures. Comprehensive phenotyping was performed identically at visit 1 (baseline visit) and after 12 weeks of the intervention (visit 3). In addition, patients received a follow-up visit one week after randomization (visit 2).

During the visit at the study center, information on current medication (according to Anatomical Therapeutic Chemical (ATC) classification system), cardiovascular risk factors and comorbidities (e.g., atrial fibrillation, coronary artery disease, chronic obstructive pulmonary disease, myocardial infarction, peripheral artery disease stroke, and venous thromboembolism) was collected through physical examination, computer-assisted interviews, anthropometric and blood pressure measurements, as well as laboratory analysis (see Supplemental Appendix for detailed information). In addition to medical-technical examinations, which were mainly focused on the cardiovascular system, blood and urine samples were taken and subsequently stored at − 80 °C for biobanking.

Transthoracic echocardiography was conducted using an iE33 echocardiography system with an S5-1 sector array transducer (Royal Philips Electronics, Amsterdam, The Netherlands). Measurements of cardiac structure and function were taken according to current guideline recommendations [11]. All datasets were digitally transferred to a server with an integrated multimodal image management system (Xcelera, Royal Philips Electronics, Amsterdam, The Netherlands) and reviewed by an experienced board-certified cardiologist in a blinded manner.

### Study endpoints

The primary endpoint of the EmDia trial was defined as the change of *E*/*E*´ ratio after 12 weeks of intervention. In this report, the following secondary and tertiary endpoints have been explored: left ventricular ejection fraction, left ventricular end-diastolic volume, left ventricular mass index, humoral biomarkers of cardiovascular disease (i.e., troponin, N-terminal pro brain natriuretic peptide (NT-proBNP), C-reactive protein), blood count (i.e., red blood cell count, leukocyte count, platelet count, hemoglobin, hematocrit, mean corpuscular hemoglobin, mean corpuscular hemoglobin concentration, mean platelet volume), vital signs (i.e., systolic and diastolic blood pressure, heart rate), and metabolism (i.e., HbA1c, body mass index, uric acid, and fatty liver index).

### Statistical analysis

All randomized subjects who received at least one dose of trial treatment and had at least one available post-baseline assessment of the primary analysis variable were included in the intention-to-treat (ITT) population (primary analysis sample). Continuous variables are presented by mean and standard deviation for normal distributions and by median and interquartile range for skewed distributions. Discrete variables are described by relative and absolute frequencies.

To account for potential differences between groups and to increase statistical power, it was pre-specified that the analysis of study endpoints would be performed by linear regression analysis with the study endpoint as the dependent variable and empagliflozin 10 mg/day versus placebo as predictor, adjusting for age, sex, and baseline value of each outcome parameter. The respective estimates provide the difference of the change scores by groups. To assess the robustness of the potential effect of empagliflozin on the primary study endpoint, sensitivity analyses were performed in the per-protocol sample and in clinically relevant subgroups: stratified by preserved and reduced left ventricular ejection fraction (≥ 55% vs. < 55%), NT-proBNP within and outside the reference range (< 125 pg/ml vs. ≥ 125 pg/ml), presence of congestive heart failure, left ventricular hypertrophy, and obesity, but also level of uric acid, eGFR, and HbA1c. Finally, mediation analysis using linear regression was performed to quantify the contribution of changes in selected biomarkers to the effect of empagliflozin on the *E*/*e*´ ratio after 12 weeks of intervention. In this study, a *P*-value < 0.05 was considered as statistically significant. Statistical analyses were performed using the R software package (Version 4.0.3).

## Results

### Cohort characteristics at baseline

Out of a total of 301 individuals screened, *N* = 144 subjects were enrolled and randomized in the EmDia trial. The analysis sample consisted of *N* = 142 patients with at least one follow-up assessment of left ventricular *E*/´ ratio. The mean age of the study cohort was 68.9 ± 7.7 years with 14.1% female subjects. The clinical characteristics of the analysis sample stratified by treatment group are displayed in Table 1. No significant differences were observed between the intervention group (empagliflozin 10 mg/day) and the control group (placebo) with regard to clinical profile including traditional cardiovascular risk factors and comorbidities. The most frequently recorded comorbidities were coronary artery disease (40.2%), followed by atrial fibrillation (30.2%) and chronic heart failure (21.1%). Among individuals with chronic heart failure, the predominant HF phenotype were HF with preserved ejection fraction (HFpEF; *n* = 21) and HF with mildly reduced ejection fraction (HFmrEF; *n* = 9); no subject suffered from HF with reduced ejection fraction (HFrEF) at baseline. HbA1c levels at baseline did not differ between groups with 7.4% (interquartile range (IQR) 7.0%/8.2%) for the placebo group and 7.3% (IQR 7.0%/7.7%) for the empagliflozin group (*P* for difference = 0.30). Regarding the primary outcome measure *E*/*e*´ ratio, a significant difference was observed at baseline: 9.06 (IQR 8.06/10.30) in patients receiving placebo compared to 9.90 (IQR 8.40/11.90) in patients receiving empagliflozin 10 mg/day (*P* = 0.031). Left ventricular ejection fraction (placebo: 57.9% ± 5.7% vs. empagliflozin: 59.2% ± 5.5%, *P* = 0.19) and NT-proBNP (placebo: 128.9 pg/ml (IQR 53.2 pg/ml /352.5 pg/ml) vs. empagliflozin: 141.7 pg/ml (IQR 59.2 pg/ml /268.2 pg/ml), *P* = 0.96) did not differ significantly between strata.

**Table 1:** Baseline characteristics of the analysis sample by intervention group

	Placebo	Empagliflozin	*P*-value
Sample size (*n*)	71	71	
Age [years] (SD)	68.5 ± 8.0	69.3 ± 7.4	0.53
Sex [female]—[%] (*n*)	12.7% (9)	15.5% (11)	0.81
Heart rate [bpm] (SD)	68.6 ± 10.4	68.3 ± 10.5	0.85
Blood pressure—Systolic [mmHg] (SD)	132.8 ± 15.9	134.5 ± 15.9	0.53
—Diastolic [mmHg] (SD)	76.1 ± 9.3	77.4 ± 9.2	0.38
Body Mass Index [kg/m^2^] (SD)	31.8 ± 4.8	31.9 ± 4.7	0.85
NT-proBNP [pg/ml] (IQR)	129 (53/353)	142 (59/268)	0.96
Traditional cardiovascular risk factors			
Arterial Hypertension—[%] (*n*)	94.4% (67)	84.5% (60)	0.099
Diabetes mellitus—[%] (*n*)	100% (71)	100% (71)	1.0
Insulin-dependent diabetes mellitus—[%] (*n*)	45.1% (32)	50.7% (36)	0.61
HbA1c [%] (IQR)	7.4 (7.0/8.2)	7.3 (7.0/7.7)	0.30
Dyslipidemia—[%] (*n*)	90.1% (64)	84.5% (60)	0.45
Family history of MI and/or stroke—[%] (*n*)	32.4% (23)	28.2% (20)	0.72
Obesity—[%] (*n*)	57.7% (41)	64.8% (46)	0.49
Smoking—[%] (*n*)	15.5% (11)	12.7% (9)	0.81
Comorbidities			
Atrial fibrillation—[%] (*n*)	29.9% (20)	23.2% (16)	0.44
Chronic kidney disease—[%] (*n*)	20.0% (14)	13.0% (9)	0.36
Chronic obstructive pulmonary disease—[%] (*n*)	5.6% (4)	7.1% (5)	0.74
Chronic heart failure—[%] (*n*)	26.8% (19)	15.5% (11)	0.15
Coronary artery disease—[%] (*n*)	44.1% (30)	35.9% (23)	0.38
History of myocardial infarction—[%] (*n*)	37.1% (26)	23.2% (16)	0.098
History of TIA/Stroke—[%] (*n*)	9.6% (7)	7.1% (5)	0.77
Peripheral artery disease—[%] (*n*)	11.9% (8)	10.1% (7)	0.79
Venous thromboembolism—[%] (*n*)	10.0% (7)	7.1% (5)	0.76
Echocardiography			
Lateral *E*/*e*´ ratio (IQR)	9.1 (8.1/10.3)	9.9 (8.4/11.9)	0.031
LVEF [%] (SD)	57.9 ± 5.7	59.2 ± 5.5	0.19
Left ventricular mass index [g/m^2.7^] (SD)	95.2 ± 25.0	94.6 ± 26.9	0.90

Absolute and relative frequency of categorical variables and mean with standard deviation (SD) or median with interquartile range (IQR) for continuous traits (dependent on distribution)

*LVEF* left ventricular ejection fraction, *MI* myocardial infarction, *NT-proBNP* N-terminal pro brain natriuretic peptide

### Effect of empagliflozin compared to placebo on left ventricular diastolic function

**Fig. 1:**
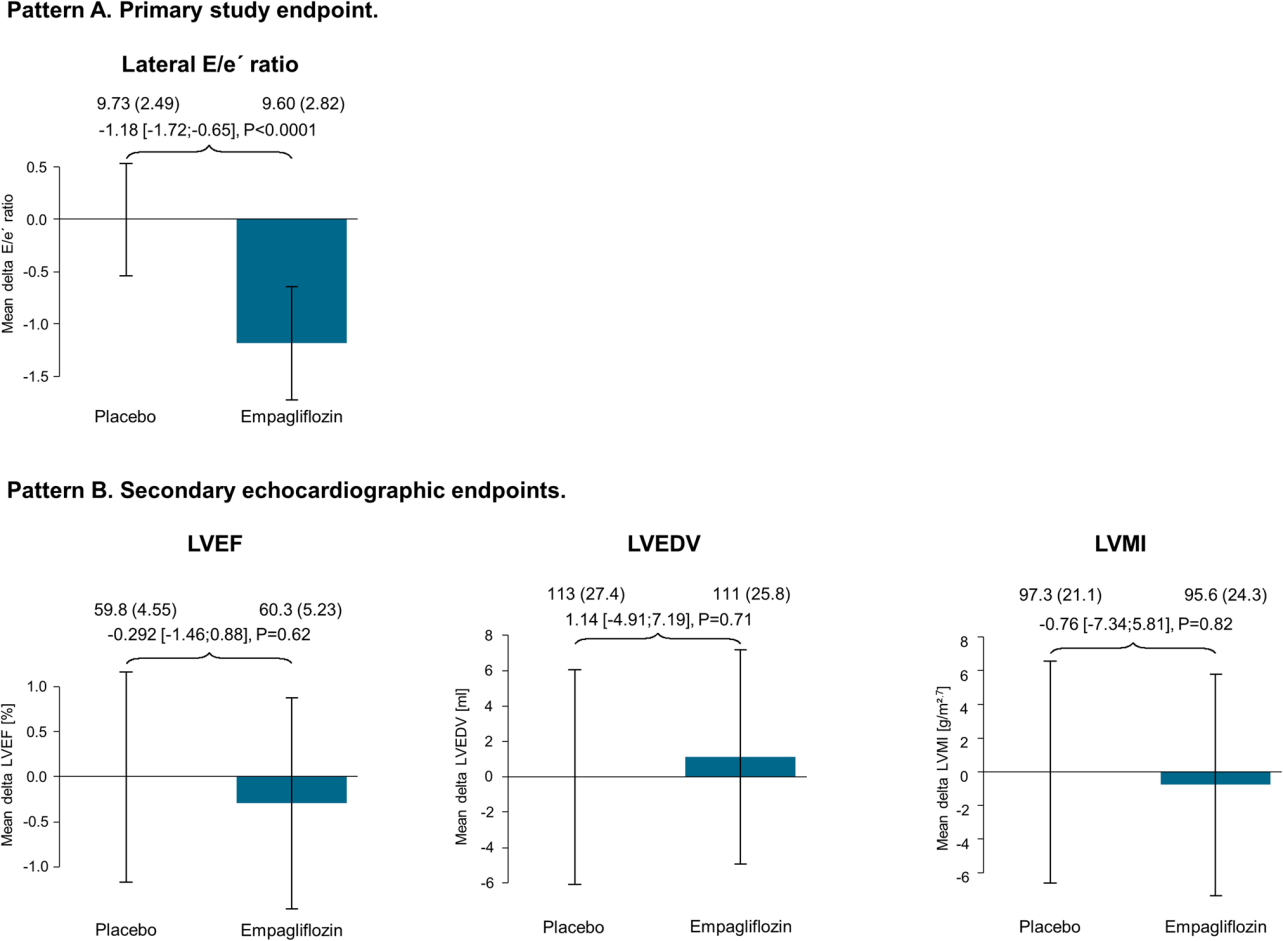
Pre-specified analysis of the effect of empagliflozin 10 mg/day versus placebo on the primary study endpoint *E*/*e*´ ratio and selected echocardiographic endpoints after 12 weeks of treatment. The figure displays the estimates of linear regression models with adjustment for age, sex, and baseline value of *E*/*e*´ ratio. The dependent variable is left ventricular *E*/*e*´ ratio after 12 weeks of intervention (Pattern A) and left ventricular ejection fraction, left ventricular end-diastolic volume and left ventricular mass index (Pattern B), respectively. The beta-estimate is given for the effect of empagliflozin (10 mg/day) versus placebo with accompanying 95% confidence interval. In addition, mean crude values (with standard deviation) of the endpoint measures are provided in the first line. Complete information on the primary outcome measure, i.e., left ventricular *E*/*e*´ ratio, was available for on *N* = 136 individuals (empagliflozin group: *N* = 67; placebo group: *N* = 69). *CI* confidence interval, *LVEDV* left ventricular end-diastolic volume, *LVEF* left ventricular ejection fraction, *LVMI* left ventricular mass index

The *E*/*e*´ ratio decreased from baseline [9.90 (IQR 8.40/11.90)] through week 1 ]9.49 (IQR 8.14/11.18)] and week 12 (9.12 (IQR 7.51/10.80)) of intervention in individuals receiving empagliflozin 10 mg/day, while no change in *E*/*e*´ ratio was observed in individuals receiving placebo. The analysis of the primary study endpoint, i.e., the change in *E*/*e*´ ratio after 12 weeks of intervention, in a linear regression analysis with *E*/*e*´ ratio at 12 weeks as dependent variable and adjustment for the covariates age, sex, and *E*/*e*´ ratio at baseline, demonstrated that empagliflozin led to a significant decrease in *E*/*e*´ ratio by − 1.18 (95% confidence interval (CI) − 1.72 to − 0.65; *P* < 0.0001) after 12 weeks of intervention compared with placebo (see Fig. 1). This result was confirmed in the analysis of the primary study endpoint in the per-protocol sample (β-estimate: − 1.17 (95% CI − 1.73 to − 0.62), *P* < 0.0001) and also in sensitivity analysis with additional adjustment for arterial hypertension (β-estimate: − 1.15 (95% CI − 1.69/− 0.61, *P* < 0.0001). When analyzing the components of *E*/*e*´ ratio separately a trend for a beneficial effect of empagliflozin on both E (β-estimate: − 0.79, 95% CI − 8.34 to 0.143; *P* = 0.06) and *e*´ (β-estimate: 0.45, 95% CI − 0.06 to 0.95; *P* = 0.088) was observed. In the placebo group, there was no significant decrease of *E*/*e*´ ratio after 12 weeks of intervention (*P* = 0.53).

Analysis of the short-term effect of empagliflozin on diastolic function after 1 week of intervention indicated a decrease in *E*/*e*´ ratio by empagliflozin compared with placebo (β-estimate: − 0.52 (95% CI − 1.10 to 0.06), although this result did not pass the threshold of statistical significance (*P* = 0.081).

### Additional study endpoints

To comprehensively evaluate the effects of empagliflozin 10 mg/day compared with placebo in individuals with type 2 diabetes mellitus, further endpoints were analyzed. First, the change of left ventricular ejection fraction, left ventricular mass index, and left-ventricular end-diastolic volume throughout the study was assessed in crude analysis. Subsequently, a linear regression analysis with adjustment for age, sex, and baseline value of the respective biomarker confirmed the absence of an impact of empagliflozin on left ventricular systolic function and left ventricular hypertrophy after 1 and 12 weeks of treatment, respectively (see Table 2). For NT-proBNP levels, a short-term effect was detected after 1 week of intervention (β-estimate: − 0.23 (95% CI − 0.30 to − 0.06), *P* = 0.009), which was not seen after 12 weeks (β-estimate: − 0.01 95% CI − 0.195 to 0.175), *P* = 0.91). A similar result was registered for the effect of empagliflozin on systolic blood pressure (β-estimate_after 1 week_: − 4.53 (95% CI − 8.13 to − 0.92), *P* = 0.015) and diastolic blood pressure (β-estimate_after 1 week_: -3.05 (95% CI − 5.05 to − 1.05), *P* = 0.0034). With regard to measures of vascular function (i.e., carotid-femoral pulse wave velocity, augmentation index, arterial stiffness index and reflection index), no statistical effect of empagliflozin compared to placebo was detectable. The ankle–brachial index was not affected by empagliflozin after 12 weeks of intervention compared to placebo (beta-estimate: − 0.0158, 95% CI − 0.0578 to 0.0261; *P* = 0.46). Furthermore, no changes were observed for relative wall thickness, troponin or heart rate under treatment.

**Table 2:** Effect of empagliflozin 10 mg/day compared to placebo on biomarkers of cardiac function, cardiac structure, circulation, metabolism and hematology after 1 and 12 weeks of intervention in individuals with type 2 diabetes mellitus

	After 1 week of treatment		After 12 weeks of treatment	
	Beta-estimate (95% CI)	*P*-value	Beta-estimate (95% CI)	*P*-value
Cardiac biomarkers				
Left ventricular ejection fraction^a^ [%]	0.4 (– 0.8; 1.6)	0.48	– 0.3 (– 1.5; 0.9)	0.62
Left ventricular end-diastolic volume^a^ [ml]	– 3.1 (– 8.1; 1.9)	0.23	1.1 (– 4.9; 7.1)	0.71
Left ventricular mass index [g/m^2.7^]	– 3.9 (– 9.9; 2.1)	0.20	– 0.8 (– 7.3; 5.8)	0.82
Relative wall thickness	– 0.005 (– 0.029; 0.02)	0.72	0.00351 (– 0.023; 0.0301)	0.80
NT-proBNP^a^ [pg/ml]	– 0.23 (– 0.30; – 0.06)	0.009	– 0.01 (– 0.195; 0.175)	0.91
Troponin^a^ [pg/ml]	0.09 (– 0.05; 0.22)	0.21	– 0.071 (– 0.188; 0.0458)	0.24
Circulation				
Blood pressure—Systolic [mmHg]	– 4.53 (– 8.13; – 0.92)	0.015	– 3.39 (– 7.37; 0.60)	0.098
Diastolic [mmHg]	– 3.05 (– 5.05; – 1.05)	0.0034	– 1.38 (– 3.35; 0.582)	0.17
Heart rate [bpm]	– 0.53 (– 2.63; 1.57)	0.62	– 0.16 (– 3.23; 2.92)	0.92
Metabolic biomarkers				
Body mass index [kg/m^2^]	– 0.42 (– 0.59; – 0.24)	< 0.0001	– 0.71 (– 0.99; – 0.44)	< 0.0001
C-reactive protein^a^ [mg/L]	0.787 (– 0.122; 0.279)	0.44	– 0.185 (– 0.459; 0.089)	0.19
Fatty liver index	– 0.516 (– 2.45; 1.29)	0.55	– 2.12 (– 4.81; 0.564)	0.12
HbA1c [%]	– 0.001 (– 0.072; 0.069)	0.97	– 0.43 (– 0.62; – 0.23)	< 0.0001
Uric acid [mg/dL]	– 1.01 (– 1.28; – 0.75)	< 0.0001	– 0.66 (– 1.01; – 2.96)	0.0005
Hematological biomarkers				
Leukocyte count [/nL]	0.39 (0.05; 0.72)	0.027	– 0.19 (– 0.58; 0.20)	0.34
Red blood cell count [/pL]	0.11 (0.04; 0.17)	0.002	0.29 (0.20; 0.38)	< 0.0001
Hemoglobin [g/dL]	0.24 (0.06; 0.42)	0.01	0.82 (0.55; 1.09)	< 0.0001
Mean corpuscular hemoglobin [pg]	– 0.21 (– 0.42; – 0.004)	0.048	– 0.13 (– 0.40; 0.14)	0.34
Mean corpuscular hemoglobin concentration [g/L]	– 0.28 (– 0.52; – 0.04)	0.026	– 0.36 (– 0.61; – 0.12)	0.005
Mean corpuscular volume [fL]	0.02 (– 0.36; 0.40)	0.92	0.50 (– 0.13; 1.12)	0.12
Hematocrit [%]	1.05 (0.45; 1.65)	0.0009	2.91 (2.09; 3.73)	< 0.0001
Thrombocyte count [/nL]	8.99 (1.22; 16.8)	0.025	– 5.34 (– 13.6; 2.94)	0.21

Linear regression with adjustment for age, sex, and baseline value of the dependent variable (as indicated in the left column) at the baseline visit; dependent variable: endpoint after 1 week and 12 weeks of treatment, respectively. Displayed is the beta-estimate with accompanying 95% confidence interval and P-value for the effect of empagliflozin 10 mg/day vs. placebo. NT-proBNP, Troponin, C-reactive protein were analyzed as dependent variable after logarithmic transformation

*CI* confidence interval

^a^Pre-specified secondary endpoint

**Table 3:** Mediation analysis of the effect of empagliflozin 10 mg/day on *E*/*e*´ ratio after 12 weeks of treatment in individuals with type 2 diabetes mellitus

	Beta-estimate_Empagliflozin 10 mg/day vs. Placebo_ [95% CI]	*P*-value
Basic model^a^	– 1.18 [– 1.72; – 0.65]	< 0.0001
+ ∆ Hemoglobin	– 0.95 [– 1.54; – 0.36]	0.0021
+ ∆ Hematocrit	– 1.04 [– 1.66; – 0.43]	0.0012
+ ∆ Estimated glomerular filtration rate	– 1.09 [– 1.63; – 0.55]	0.00013
+ ∆ Body mass index	– 1.11 [– 1.69; – 0.52]	0.00031
+ ∆ Systolic blood pressure	– 1.14 [– 1.68; – 0.59]	< 0.0001
+ ∆ Diastolic blood pressure	– 1.16 [– 1.71; – 0.61]	< 0.0001
+ ∆ HbA1c	– 1.17 [– 1.74; – 0.60]	0.0001
+ ∆ NT-proBNP	– 1.19 [– 1.74; – 0.64]	< 0.0001
+ ∆ Uric acid	– 1.19 [– 1.75; – 0.63]	< 0.0001
Full model^b^	– 0.77 [– 1.52; – 0.02]	0.046

The table displays linear regression models with *E*/*e*´ ratio after 12 weeks of intervention as dependent variable. The beta-estimate provided illustrates the effect of empagliflozin 10 mg/day compared to placebo

In order to decipher the mediation of the effect of empagliflozin on diastolic function via distinct pathways, estimates for empagliflozin versus placebo have been first adjusted for the delta in the following variables between baseline and 12 weeks of intervention in separate models: body mass index (BMI), systolic blood pressure (SBP), diastolic blood pressure (DBP), renal function as estimated by CKD-EPI formula as estimated glomerular filtration rate (eGFR), HbA1c, NT-proBNP, uric acid, hematocrit and hemoglobin

*BNP* brain natriuretic peptide

^a^The basic model is adjusted for age, sex, and *E*/*e*´ ratio at baseline. In the case of effect modification introduced by a single covariate or the sum of covariates, a reduction of the estimate of empagliflozin 10 mg/day versus placebo would be expected

^b^The joint contribution of all factors to the impact of empagliflozin on *E*/*e*´ ratio has been evaluated in a full model including all parameters as independent predictors

Due to the known impact of empagliflozin on body weight, further metabolic biomarkers were explored. Multivariable regression analysis revealed a significant effect of empagliflozin 10 mg/day versus placebo on body mass index (β-estimate: − 0.71 (95% CI − 0.99 to − 0.44; *P* < 0.0001), HbA1c (β-estimate: − 0.43 (95% CI − 0.62 to − 0.23), *P* < 0.0001), and uric acid (β-estimate: − 0.66 (95% CI − 1.01 to − 2.96), *P* = 0.0005) after 12 weeks of intervention. No significant effect of empagliflozin was found with respect to C-reactive protein and fatty liver index levels.

Last, the effect of empagliflozin on hematological biomarkers was analyzed. No significant effect of empagliflozin on leukocyte and platelet count was found, whereas a highly significant increase in red blood cell count was detected (β-estimate: 0.29 (95% CI 0.20–0.38), *P* < 0.0001). In addition, mean corpuscular hemoglobin concentration was reduced by empagliflozin (β-estimate: − 0.36 (95% CI − 0.61 to − 0.12), *P* = 0.005), whereas hematocrit (β-estimate: 2.91 (95% CI 2.09–3.73; *P* < 0.0001) and level of hemoglobin (β-estimate: 0.82 (95% CI 0.55–1.09), *P* < 0.0001) were increased by 12 weeks of therapy with empagliflozin.

### Sensitivity and mediation analyses for the change in left ventricular diastolic function

**Fig. 2:**
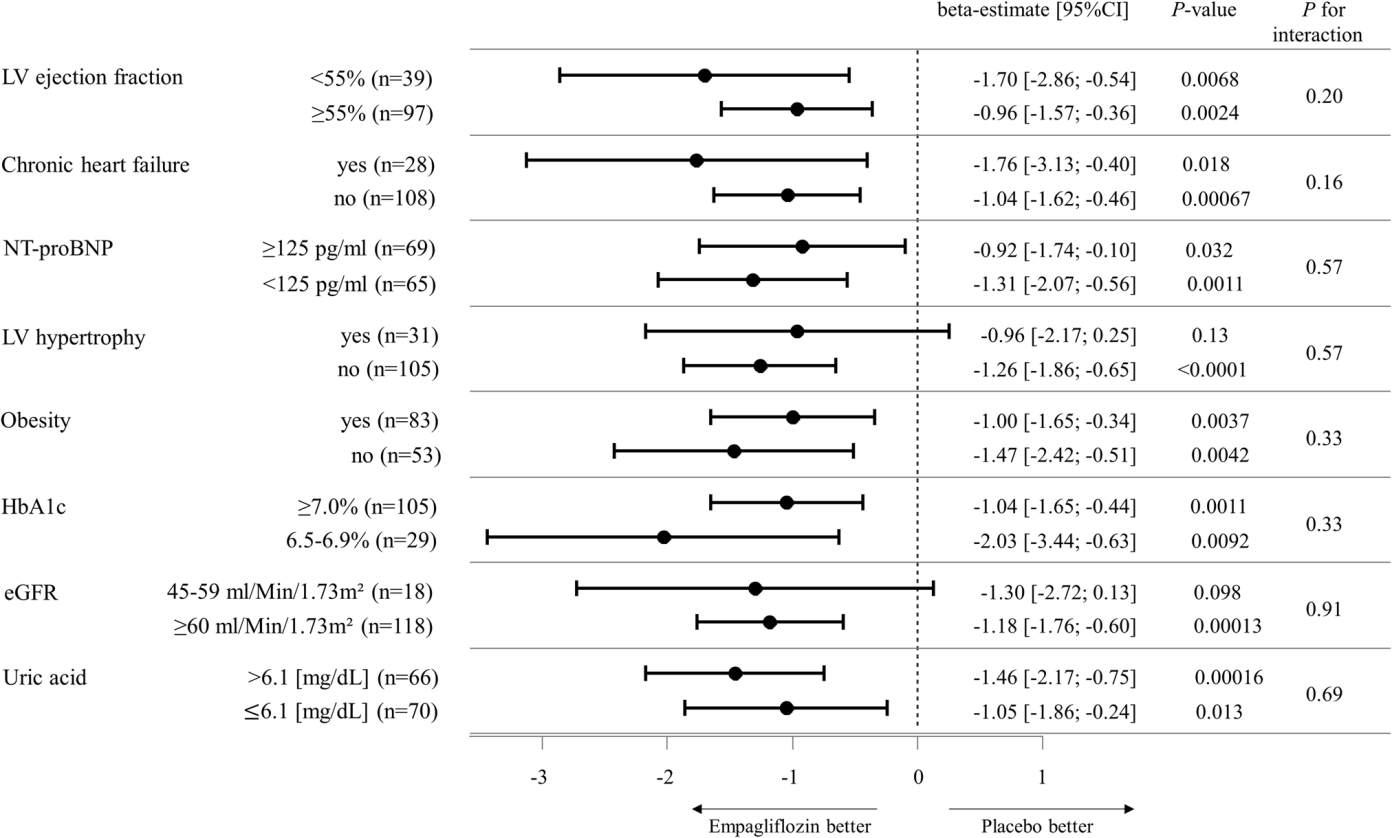
Effect of empagliflozin 10 mg/day vs. placebo on the primary study endpoint after 12 weeks of intervention in clinically-relevant subgroups. Beta-estimates for the effect of empagliflozin 10 mg/day compared to placebo on *E*/*e*´ ratio after 12 weeks of intervention are calculated by linear regression model with adjustment for age, sex, and baseline value of *E*/*e*´ ratio stratified by subgroups. Subgroups of eGFR and HbA1c were analyzed as predefined in the study protocol. HbA1c and uric acid were stratified by median, whereas LV ejection fraction was stratified by 55% according to distribution and NT-proBNP was stratified according to its use in the diagnosis of heart failure according to current guidelines. The squares with horizontal lines represent beta-estimates and corresponding confidence intervals. *BNP* brain natriuretic peptide, *CI* confidence interval, *eGFR* estimated glomerular filtration rate, *LV* left ventricular

In a next step, sensitivity analysis in clinically relevant subgroups has been carried out (Fig. 2). Of clinical relevance, stratified analysis by preserved versus reduced left ventricular ejection fraction, NT-proBNP in- and outside the reference range, presence of congestive heart failure, of obesity or left ventricular hypertrophy, levels of eGFR, HbA1c and uric acid demonstrated consistency and robustness of the effect of empagliflozin 10 mg/day compared with placebo on left ventricular *E*/*e*´ ratio across subgroups. For the subgroup with congestive heart failure, a mean LVEF of 54.6% ± 5.6% (placebo group) and 56.2% ± 7.0% (intervention group) was documented at baseline examination (lowest LVEF: 43.2%), indicating the predominant prevalence of heart failure with preserved ejection fraction in the cohort.

To decipher the contribution of the systemic effects of empagliflozin on the change in left ventricular diastolic function, a mediation analysis for the effect of 12-week empagliflozin on *E*/*E*´ ratio was carried out. As illustrated in Table 3, the contribution of the following parameters to the change in *E*/*E*' ratio was investigated: body mass index, systolic and diastolic blood pressure, eGFR, HbA1c, NT-proBNP, uric acid, hematocrit, and hemoglobin. The strongest effects on the change in left ventricular diastolic function were determined by the effect of empagliflozin on hemoglobin, hematocrit, and glomerular filtration rate. Overall, approximately a third of the reduction of *E*/*e*´ ratio by empagliflozin was found to be explained via the impact of empagliflozin on all variables explored in the analysis (β–estimate_fully adjusted_: − 0.77 (95% CI − 1.52 to − 0.02), *P* = 0.046).

## Discussion

The present study investigated the effects of the SGLT2 inhibitor empagliflozin in patients with diastolic dysfunction and type 2 diabetes mellitus. The results indicate a significant improvement in diastolic function as measured by left ventricular *E*/*e*´ ratio after 12 weeks of therapy with empagliflozin 10 mg/day compared to placebo as an adjunct to standard therapy, which was consistent across subgroups. The change in *E*/*e*´ ratio was accompanied by significant effects of empagliflozin on metabolic and hematologic biomarkers in this cohort with predominantly preserved left ventricular ejection fraction, which in turn explain approximately one-third of the beneficial effect of the drug on left ventricular diastolic function.

The results of the EmDia trial add to the growing body of evidence supporting a positive effect of empagliflozin on cardiovascular health [2]. Although these data provide clear evidence of an effect on cardiovascular disease, the underlying mechanisms still need to be defined. With regard to echocardiographic studies, a recent meta-analysis that pooled data from very small study samples revealed that empagliflozin may have a beneficial effect on *E*/*e*´ ratio [12]. Interestingly, data supporting a positive impact of SGLT2 inhibitors on the *E*/*e*´ ratio are more consistent for individuals at risk of developing heart failure (i.e., heart failure at American Heart Association (AHA) stage A/B) [13–15] as compared with individuals with symptomatic heart failure of AHA stage C [16–18]. Recent data from a subgroup analysis of the Empire HF trial showed that empagliflozin reduced left ventricular and left atrial volumes in patients with heart failure and reduced ejection fraction, which is consistent with findings from another randomized trial in non-diabetic subjects with heart failure and reduced ejection fraction [19, 20]. In contrast, the EMPA-HEART CardioLink-6 trial did not show a positive impact of empagliflozin on left ventricular diastolic function in individuals with diabetes mellitus and coronary artery disease [21]. The improvement in diastolic function found in the EmDia trial is also supported by recent data establishing a reduction in pulmonary pressures measured with an implanted pulmonary artery pressure sensor by empagliflozin compared to placebo [22]. Given the known beneficial effect of mineralocorticoid receptor antagonists on *E*/*e*´ ratio[23] and subsequent reduction in hospitalization for heart failure[24], the reduction in *E*/*e*´ ratio observed in the EmDia trial is of specific interest in the context of the beneficial effect of empagliflozin in patients with heart failure and preserved ejection fraction with and without diabetes mellitus [25].

The transient reduction in systolic and diastolic blood pressure after one week of therapy supports the hypothesis that part, and in particular the early, cardiovascular effects of empagliflozin may be mediated via an improvement in ventricular loading through a reduction in afterload that is likely secondary to the diuretic effects of the drug [26]. In the present study results, the improvement in ventricular loading was reflected by the short-term decrease in NTproBNP and the diuretic effect of the drug was mirrored by the increase in hematocrit, which was also observed in other trials such as the EMPA-HEART CardioLink-6 study [13]. In addition, effects of empagliflozin on hematological and metabolic biomarkers were encountered in the present study: The impact of empagliflozin on red blood cells found in the EmDia trial is likely to be explained by the earlier reported SGLT2 inhibitor-mediated increase in erythropoietin production, a change in red blood cell morphology and iron utilization [27]. The reduction in plasmatic levels of uric acid under treatment is of clinical relevance given abundant evidence on the prognostic value of uric acid [28].

The improvement in diastolic function found in the EmDia trial provides new insights relevant in context of the results of the recently published EMPEROR-PRESERVED clinical trial investigating the efficacy and safety of empagliflozin in individuals with heart failure and preserved ejection fraction (defined by a left ventricular ejection fraction > 40%) independent of the presence of diabetes mellitus [5]. Since no interaction for improvement in diastolic function was found with the presence of congestive heart failure with LVEF > 40% in the EmDia trial, it seems likely that empagliflozin will also improve diastolic dysfunction in patients with heart failure and preserved ejection fraction irrespective of the presence of diabetes mellitus. Further research is needed, however, to clarify whether empagliflozin emerges as novel treatment approach in chronic heart failure independent of the heart failure phenotype.

### Strengths and limitations

The major strength of the present study is the well-phenotyped cohort, which was studied in a dedicated study center by trained staff in a highly standardized setting minimizing variability of data assessment at high accuracy and reproducibility. However, several limitations should be noted when interpreting the study results. The assessment of diastolic function was limited to left ventricular *E*/*e*´ ratio, which precluded consideration of other markers for cardiac function that have been reported to predict cardiovascular outcome, such as left atrial volume index or cardiac strain [29]. Since left ventricular *E*/*e*´ ratio is known to predict cardiovascular outcome [30], the demonstrated effects of empagliflozin on diastolic function are likely to be clinically relevant. As patients were recruited in a clinically stable condition, findings cannot be translated to the setting of acute decompensated heart failure. Due to limited sample size in the subsample of patients with HF, analysis stratified by HF phenotypes could not be performed. Finally, only one dosage of empagliflozin was investigated. However, a dose-dependent effect on diastolic function seems unlikely, as the effects of empagliflozin on cardiovascular outcome did not differ substantially between doses [31].

## Conclusions

The results of the present EmDia trial demonstrated that empagliflozin 10 mg/day improved diastolic function in patients with type 2 diabetes mellitus and elevated left ventricular end-diastolic pressure within 12 weeks of treatment. The beneficial effect of empagliflozin was consistent across all subgroups and also occurred in subjects with heart failure and preserved ejection fraction, supporting the positive effect of empagliflozin reported in the literature regarding the treatment of patients with heart failure and including patients with heart failure with preserved ejection fraction. Because the identified positive hemodynamic, metabolic, and hematological effects of empagliflozin explain only part of its effect on cardiac function, future studies will be important to identify to what extent this mechanism contributes to improved clinical outcome in subjects with heart failure.

## Supplementary Information

Below is the link to the electronic supplementary material.

Supplementary file1 (DOCX 144 KB)

## Data Availability

This project constitutes a major scientific effort with high methodological standards and detailed guidelines for analysis and publication. Data are not made available for the scientific community outside the established and controlled workflows and algorithms. To meet the general idea of verification and reproducibility of scientific findings, we offer access to data at the local database in accordance with the ethics vote on request (contact: Prof. Dr. Philipp Wild (PI), philipp.wild@unimedizin-mainz.de).

## Declarations

### Collaborators of the study

Dr. Jürgen H. Prochaska, Dr. Andreas Schulz, Dr. Natalie Arnold, Dr. Felix Müller, Dr. Marc William Heidorn, Rieke Baumkötter, Dr. Daniela Zahn, Dr. Thomas Koeck, Dr. Sven-Oliver Tröbs, Dr. Karl J. Lackner, Dr. Andreas Daiber, Dr. Harald Binder, Dr. Sanjiv J. Shah, Dr. Tommaso Gori, Dr. Thomas Münzel, Dr. Philipp S. Wild, Dr. Steffen Rapp; Interdisciplinary Center for Clinical Trials (IZKS) Mainz (i.e., Dr. Silke Warnke, Dr. Kai Kronfeld, Dr. Christian Ruckes, Christoph Medler).
