# Supplementary material for: Effects of empagliflozin on left ventricular diastolic function in addition to usual care in individuals with type 2 diabetes mellitus—results from the randomized, double-blind, placebo-controlled EmDia trial
Source: Clin Res Cardiol. 2023 Feb 10;112(7):911–22. doi: 10.1007/s00392-023-02164-w (PMC10293456; doi:10.1007/s00392-023-02164-w)
Supplement: Supplementary file 1 — Supplementary file1 (DOCX 144 KB) [file 392_2023_2164_MOESM1_ESM.docx]

**SUPPLEMENTAL APPENDIX**

**Effects of empagliflozin on left ventricular diastolic function in addition to usual care in individuals with type 2 diabetes mellitus –**

**Results from the randomized, double-blind, placebo-controlled EmDia trial**

Jürgen H. Prochaska, MD^1,2,3^, Claus Jünger, MD, MSan^1,4^, Andreas Schulz, PhD^1^, Natalie Arnold, MD^1,6^, Felix Müller, MD^1,3^, Marc William Heidorn, MD^1,3^, Rieke Baumkötter, BA^1^, Daniela Zahn, PhD^1^, Thomas Koeck, PhD^1^, Sven-Oliver Tröbs, MD^1,3^, Karl J. Lackner, MD^7,3^, Andreas Daiber, PhD^8,3^, Harald Binder, PhD^5^, Sanjiv J. Shah, MD^9^, Tommaso Gori, MD, PhD^8,3^, Thomas Münzel, MD^8,3^, Philipp S. Wild, MD, MSc^1,2,3,10^

^1^ Preventive Cardiology and Preventive Medicine, Department of Cardiology, University Medical Center of the Johannes Gutenberg-University Mainz, Mainz, Germany;

^2^ Center for Thrombosis and Hemostasis (CTH), University Medical Center of the Johannes Gutenberg-University Mainz, Mainz, Germany;

^3^ German Center for Cardiovascular Research (DZHK), Partner Site Rhine Main, Mainz, Germany

^4^ Department of Psychosomatic Medicine and Psychotherapy, University Medical Center of the Johannes Gutenberg-University Mainz, Mainz, Germany;

^5^ Institute of Medical Biometry and Statistics, University of Freiburg, Freiburg, Germany;

^6^ University Heart and Vascular Center Hamburg, Department of Cardiology, Hamburg, Germany;

^7^ Institute for Clinical Chemistry and Laboratory Medicine, University Medical Center of the Johannes Gutenberg-University Mainz, Mainz, Germany;

^8^ Department of Cardiology, University Medical Center of the Johannes Gutenberg-University Mainz, Mainz, Germany;

^9^ Division of Cardiology, Department of Medicine, Northwestern University Feinberg School of Medicine, Chicago, IL, USA.

^10^ Systems Medicine, Institute for Molecular Biology (IMB), Mainz, Germany.

**Table of Contents**

Page

[**Supplemental methods.** 3](#_Toc66278936)

[**Supplemental Table 1.** Distribution of laboratory biomarkers and medication in the analysis sample by intervention group. 5](#_Toc66278937)

[**Supplemental Table 2.** Baseline characteristics of the per-protocol sample. 7](#_Toc66278938)

[**Supplemental Figure 1.** Consort Flow Diagram. 8](#_Toc66278939)

[**Supplemental Figure 2.** Effect of empagliflozin (10mg/day) versus placebo on E/e´ ratio after 1 and 12 weeks of intervention in the per-protocol sample. 9](#_Toc66278940)

# **Supplemental methods.**

*Inclusion and exclusion criteria of the EmDia study*

Key inclusion criteria

• Diagnosis of type 2-diabetes mellitus with stable glucose-lowering background therapy and/or dietetic treatment for at least 12 weeks

• In subjects without glucose-lowering background therapy: the application of Metformin was considered to be unsuitable due to drug intolerance

• HbA1c level of ≥6.5% and ≤10.0% for subjects on antidiabetic background therapy or HbA1c level of ≥6.5% and ≤9.0% for drug-naïve subjects with dietetic treatment

• Diastolic cardiac dysfunction E/E’ ≥8

• Age 18 – 84 years

• Body Mass Index ≤ 45 kg/m²

*Note:* During the conduct of the study, the threshold of HbA1c was changed from 7.0% to 6.5%. Therefore, analysis of the subgroup of individuals with a baseline value of HbA1c of ≥6.5% to 7.0% was introduced as additional secondary endpoint to the study protocol.

Key exclusion criteria

• Pretreatment with empagliflozin or other SGLT2 inhibitor within the last 3 months

• Impaired renal function, defined as eGFR <45 ml/min/1.73 m² of body-surface-area or end-stage renal failure or dialysis

• Pretreatment with known inducers of UGT enzymes

• Uncontrolled hyperglycemia with a glucose level > 240 mg/dl (>13.3 mmol/L) after an overnight fast

• Severe hepatic dysfunction, defined by serum levels of either SGPT, SGOT, or alkaline phosphatase above 3 x upper limit of normal

• Acute urinary tract infection

• Known acute genital infection

• Symptomatic hypotension

• Hematocrit above the upper limit of the reference range

• Hypoglycemic tendencies

• Severe PAD (Fontaine classification Stage IIb - IV)

• Medical history of cancer and/or treatment for cancer within the last 5 years (subjects with basalioma can be included in the study)

• Medical history of pancreatitis or surgery on pancreas

• Known ketoacidosis (in the past)

• NYHA classification III – IV

• Acute coronary syndrome, stroke or TIA within the last 2 months

• Planned cardiac surgery or angioplasty within 3 months

*Note:* During the conduct of the study, the threshold of eGFR was changed from <60 ml/min/1.73 m² to <45 ml/min/1.73 m². Therefore, analysis of the subgroup of individuals with a baseline value of eGFR from 45 to 60 ml/min/1.73m² was introduced as additional secondary endpoint to the study protocol.

*Definition of traditional cardiovascular risk factors*

Positive family history of stroke or myocardial infarction, obesity, smoking, diabetes mellitus, dyslipidemia and arterial hypertension were classified as traditional cardiovascular risk factors. Arterial hypertension was defined as mean systolic blood pressure of ≥140mmHg or a mean diastolic blood pressure of ≥90mmHg (mean value of the 2nd and 3rd standardized blood pressure measurement after 8 and 11 minutes of rest, respectively) or intake of antihypertensive medication in the last two weeks. Obesity was defined as a body-mass index ≥30 kg/m². Individuals were classified as smokers (daily smokers ≥1 cigarette/day and occasional smokers <1 cigarette/day), former smokers and non-smokers (never smoked). Dyslipidemia and arterial hypertension were defined in individuals with diagnosis by a physician. Dyslipidemia was additionally defined as low-density lipoprotein/high-density lipoprotein ratio of >3.5 or triglycerides level ≥150mg/dl or intake of lipid modifying agents (ATC-Code C10). Positive family history was defined as myocardial infarction and/or stroke of male first-degree relatives until the age of 60 years for female first-degree relatives until the age of 65 years.

*Assessment of comorbidities*

The presence of comorbidities, i.e. atrial fibrillation, chronic kidney disease, chronic obstructive pulmonary disease, congestive heart failure, coronary artery disease, history of myocardial infarction, history of stroke and/or transient ischemic attack, peripheral artery disease and venous thromboembolism, were assessed via a standardized computer-assisted interview by trained staff. In addition, medical records – if available for review – have been screened.

*Echocardiographic examination*

Information on cardiac function was assessed in apical four-chamber view: Pulsed wave Doppler imaging was performed with the sample volume between the opened mitral leaflets tips to assess early diastolic peak velocity (E). Early longitudinal peak diastolic velocity (E’) was determined at the lateral mitral annulus by pulsed wave tissue Doppler imaging. Left ventricular ejection fraction was quantified using the modified Simpson method and left ventricular mass index (LVMi) was calculated using the cube formula.

*Fatty liver index*

The fatty liver index was calculated based upon the variables body mass index, waist circumference, gamma-glutamyl-transferase, and concentration of triglycerides as recently recommended.^1^

**Statistical considerations**

*Sample size estimation*

For the primary study end point of the EmDia study, i.e., the change in left ventricular E/e' ratio after 12 weeks of treatment, a minimum clinically relevant difference of 1.5 with a standard deviation of 3.46 was assumed for case number planning. The standard deviation estimate was derived based on data from diabetic patients in the population-based Gutenberg Health Study (total N=15,010 ^2^). Assuming a minimum effect size of 1.5 and a standard deviation of 3.46, it was calculated that for a two-sided t test at a significance level of 0.05 and a statistical power of 80%, n=67 subjects per group were required to reject the null hypothesis (i.e., no lack of difference in left ventricular E/e' ratio between the 2 treatment groups). Considering a drop-out rate of 7.5% before visit 1 and an additional drop-out rate of 7.5% subsequent to visit 1 (until 12-week follow-up), a total sample size of n=158 with n=79 per arm would be required.

*Statistical analysis*

It was predefined that the primary endpoint will be compared between treatment groups at a two-sided significance level of alpha=0.05 by using a linear regression model with left ventricular E/e´ ratio at 12 weeks as dependent variable and with treatment as a covariate and adjustment for baseline left ventricular E/e´ ratio and sex. The rationale for the pre-specified adjusted analysis in the randomized controlled EmDia trial was chosen to maximize statistical power.^3^ Furthermore, it was defined that (i) the analysis of the primary endpoint is confirmatory and all other analyses are of explorative nature, and (II) that no correction for multiple hypothesis testing will be made.

# **Supplemental Table 1.** Distribution of laboratory biomarkers and medication in the analysis sample by intervention group.

|  | Placebo | Empagliflozin 10mg/day | *P*-value |
| --- | --- | --- | --- |
| *Medication* |  |  |  |
| Antidiabetic medication |  |  |  |
| Insulin (A10A) – [%] (n) | 45.1% (32) | 50.7% (36) | 0.61 |
| Antidiabetics excl. insulin (A10B) – [%] (n) | 87.3%% (62) | 88.7% (63) | 1.00 |
| Biguanides (A10BA)– [%] (n) | 60.6% (43) | 67.6% (48) | 0.48 |
| Sulfonylurea (A10BB)– [%] (n) | 7.0% (5) | 1.4% (1) | 0.21 |
| α-glucosidase inhibitors (A10BF) – [%] (n) | 1.4% (1) | 0 (0) | 1.00 |
| Thiazolidinediones (A10BG) – [%] (n) | 1.4% (1) | 0 (0) | 1.00 |
| DPP-4 inhibitors (A10BH) – [%] (n) | 21.1% (15) | 14.1% (10) | 0.38 |
| GLP-1 receptor agonists (A10BJ) – [%] (n) | 4.2% (3) | 9.9% (7) | 0.33 |
| ACE-inhibitor / ARB (C09) – [%] (n) | 91.5% (65) | 81.7% (58) | 0.14 |
| Antithrombotic therapy (B01) – [%] (n) | 76.1% (54) | 76.1% (54) | 1.00 |
| Beta blockers (C07) – [%] (n) | 57.7% (41) | 56.3% (40) | 1.00 |
| Calcium channel blockers (C08) – [%] (n) | 31.0% (22) | 33.8% (24) | 0.86 |
| Cardiac glycosides (C01A) – [%] (n) | 2.8% (2) | 0% (0) | 0.50 |
| Diuretics (C03) – [%] (n) | 39.4% (28) | 31.0% (22) | 0.38 |
| Lipid-modifying medication (C10) – [%] (n) | 78.9% (56) | 77.5% (55) | 1.00 |
|  |  |  |  |
| *Laboratory biomarkers* |  |  |  |
| NT-proBNP (IQR) | 128.9  (53.2/352.5) | 141.7  (59.2/268.2) | 0.96 |
| Troponin (IQR) | 3.7 (1.6/8.1) | 4.0 (2.6/6.6) | 0.47 |
| C-reactive protein (IQR) | 1.9 (1.1/3.6) | 2.2 (1.1/3.9) | 0.79 |
| eGFR [ml/Min/1.73m²] (SD) | 77.1±12.7 | 75.8±14.8 | 0.58 |
| Blood count |  |  |  |
| Leukocyte count [/nL] (IQR) | 7.18 (5.99/8.47) | 7.02 (6.18/8.58) | 0.74 |
| Red blood cell count [/pL] (SD) | 4.65±0.38 | 4.68±0.40 | 0.63 |
| Hemoglobin [g/dL] (SD) | 14.1±1.1 | 14.2±1.2 | 0.55 |
| Mean corpuscular hemoglobin [pg] (SD) | 30.5±1.8 | 30.5±1.5 | 0.99 |
| Mean corpuscular hemoglobin  concentration [g/L] (SD) | 33.7±0.9 | 33.5±0.9 | 0.25 |
| Mean corpuscular volume [fL] (SD) | 90.5±4.6 | 91.0±4.0 | 0.48 |
| Hematocrit [%](SD) | 42.0±2.8 | 42.6±3.3 | 0.27 |
| Thrombocyte count [/nL] (IQR) | 213 (178/245) | 229 (184/247) | 0.30 |
| (continued) | Placebo | Empagliflozin 10mg/day | *P*-value |
| Metabolism |  |  |  |
| LDL cholesterol (SD) [mg/dL] | 97.3±30.4 | 97.8±34.8 | 0.94 |
| HDL cholesterol (IQR) [mg/dL] | 42.0 (38.0/48.0) | 44.0 (38.0/52.8) | 0.29 |
| Triglycerides (IQR) [mg/dL] | 150.0  (97.8/196.0) | 128.0  (103.2/171.0) | 0.16 |
| Gamma- glutamyltransferase (IQR) [U/l] | 30.0 (22.0/50.0) | 30.0 (22.0/54.8) | 0.70 |
| Fatty liver index (IQR) | 86.3 (60.5/95.8) | 86.0 (67.9/93.2) | 0.78 |
| Uric acid (SD) [mg/dL] | 6.49±1.59 | 6.21±1.36 | 0.27 |

Dependent on distribution, linear trait values are presented as mean ± standard deviation or as median with interquartile range (25^th^/75^th^ percentile). Information on medication was derived from personal computer-assisted interview during the baseline visit in the study center with subsequent coding according to the anatomical therapeutic chemical (ATC) classification system of drugs. ATC codes for the categories of medication are displayed in brackets accompanying the drug’s name. The 2009 CKD-EPI equation was used to assess renal function (eGFR).^4^ ARB, angiotensin I receptor blocker; BNP, brain natriuretic peptide; excl, excluding; HDL, high density lipoprotein; IQR, interquartile range; LDL, low density lipoprotein; SD, standard deviation.

# **Supplemental Table 2.** Baseline characteristics of the per-protocol sample.

|  | Placebo | Empagliflozin  10mg/day | *P*-value |
| --- | --- | --- | --- |
| Sample size (n) | 66 | 66 |  |
| Age [years] (SD) | 68.2±7.9 | 69.3 +/- 7.5 | 0.40 |
| Sex [female] – [%] (n) | 12.1% (8) | 16.7% (11) | 0.62 |
| Heart rate [bpm] (SD) | 68.9 +/- 10.4 | 67.6 +/- 10.3 | 0.50 |
| Blood pressure – Systolic [mmHg] (SD) | 133.2 +/- 15.8 | 134.9 +/- 16.1 | 0.53 |
| – Diastolic [mmHg] (SD) | 76.0 +/- 9.1 | 77.4 +/- 9.3 | 0.37 |
| Body Mass Index [kg/m²] (SD) | 31.9 +/- 4.9 | 31.9 +/- 4.8 | 0.95 |
| Traditional cardiovascular risk factors |  |  |  |
| Arterial Hypertension – [%] (n) | 93.9% (62) | 84.8% (56) | 0.16 |
| Diabetes mellitus – [%] (n) | 100% (66) | 100% (61) | 1.00 |
| Insulin-dependent diabetes mellitus – [%] (n) | 45.5% (30) | 48.5% (32) | 0.86 |
| HbA1c [%] (IQR) | 7.40 (7.00/8.20) | 7.30 (6.99/7.70) | 0.21 |
| Dyslipidemia – [%] (n) | 90.9% (60) | 83.3% (55) | 0.30 |
| Family history of MI and/or stroke – [%] (n) | 34.8% (23) | 30.3% (20) | 0.71 |
| Obesity – [%] (n) | 57.6% (38) | 63.6% (42) | 0.59 |
| Smoking – [%] (n) | 15.2% (10) | 12.1% (8) | 0.80 |
| Comorbidities |  |  |  |
| Atrial fibrillation – [%] (n) | 30.2% (19) | 25.0% (16) | 0.56 |
| Chronic kidney disease – [%] (n) | 20.0% (13) | 14.1% (9) | 0.48 |
| Chronic obstructive pulmonary disease – [%] (n) | 6.1% (4) | 7.7% (5) | 0.74 |
| Congestive heart failure – [%] (n) | 28.8% (19) | 15.2% (10) | 0.092 |
| Coronary artery disease – [%] (n) | 44.4% (28) | 36.7% (22) | 0.46 |
| History of myocardial infarction – [%] (n) | 36.9% (24) | 21.9% (14) | 0.082 |
| History of TIA/Stroke – [%] (n) | 10.6% (7) | 7.7% (5) | 0.76 |
| Peripheral artery disease – [%] (n) | 11.3% (7) | 7.8% (5) | 0.56 |
| Venous thromboembolism – [%] (n) | 10.8% (7) | 7.7% (5) | 0.76 |

Absolute and relative frequency of categorical variables and mean with standard deviation (SD) or median with interquartile range (IQR) for continuous traits (dependent on distribution). MI, myocardial infarction.

# **Supplemental Figure 1.** Consort Flow Diagram.

**
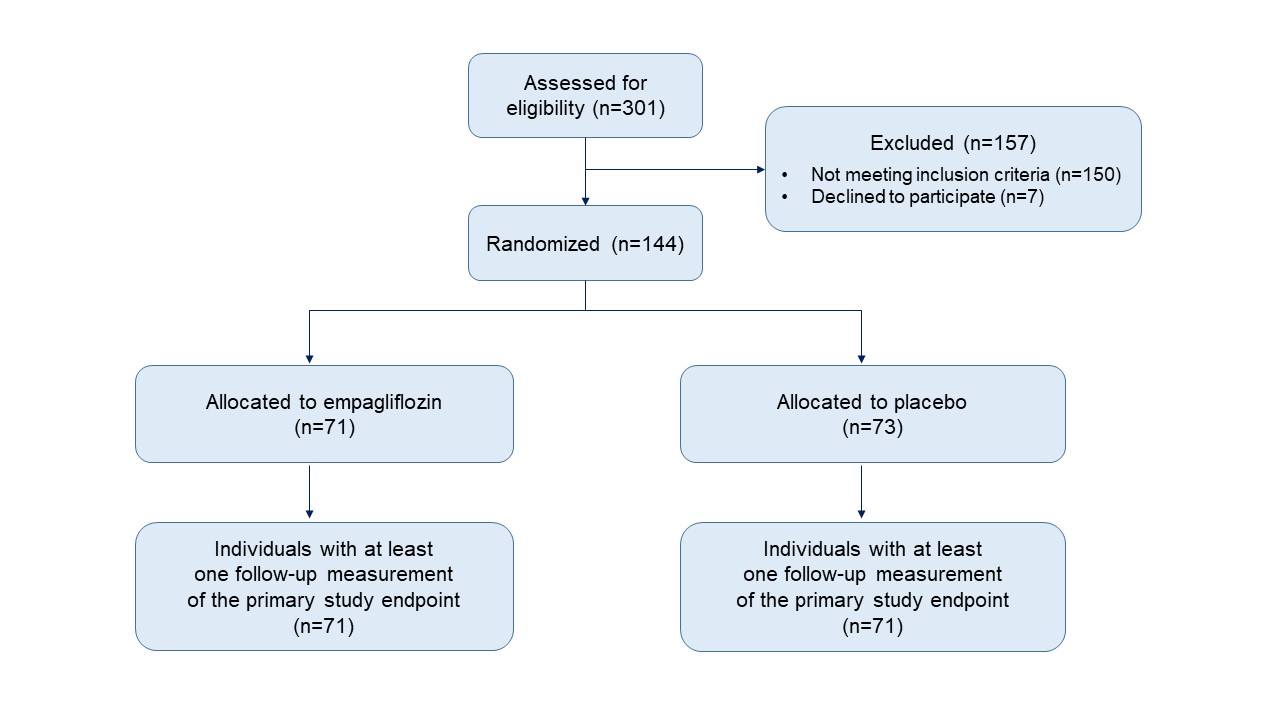
**

# **Supplemental Figure 2.** Effect of empagliflozin 10mg/day versus placebo on E/é´ ratio after 1 and 12 weeks of intervention in the per-protocol sample.


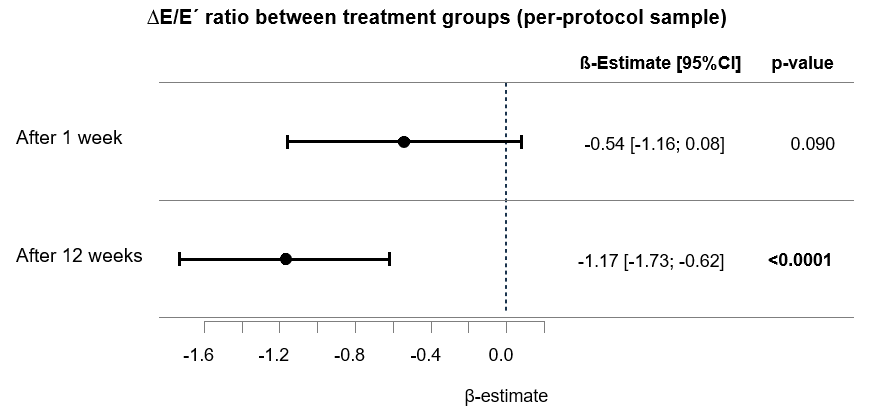


Linear regression model with adjustment for age, sex, and E/e´ ratio at baseline. Dependent variable: left ventricular E/e´ ratio after 1 and 12 weeks of intervention, respectively. Predictor: β-estimate with 95% confidence interval (CI) is provided for empagliflozin (10mg/day) versus placebo.

**References**

1. Otgonsuren M, Estep MJ, Hossain N, Younossi E, Frost S, Henry L, Hunt S, Fang Y, Goodman Z and Younossi ZM. Single non-invasive model to diagnose non-alcoholic fatty liver disease (NAFLD) and non-alcoholic steatohepatitis (NASH). *J Gastroenterol Hepatol*. 2014;29:2006-13.

2. Wild PS, Zeller T, Beutel M, Blettner M, Dugi KA, Lackner KJ, Pfeiffer N, Münzel T and Blankenberg S. [The Gutenberg Health Study]. *Bundesgesundheitsblatt Gesundheitsforschung Gesundheitsschutz*. 2012;55:824-9.

3. Yu LM, Chan AW, Hopewell S, Deeks JJ and Altman DG. Reporting on covariate adjustment in randomised controlled trials before and after revision of the 2001 CONSORT statement: a literature review. *Trials*. 2010;11:59.

4. Levey AS, Stevens LA, Schmid CH, Zhang YL, Castro AF, 3rd, Feldman HI, Kusek JW, Eggers P, Van Lente F, Greene T and Coresh J. A new equation to estimate glomerular filtration rate. *Ann Intern Med*. 2009;150:604-12.
